# Looking deeper into the algal sisters of land plants

**DOI:** 10.1371/journal.pbio.3004050

**Published:** 2026-11-04

**Authors:** Susana M. Coelho

**Affiliations:** Department of Algal Development and Evolution, Max Planck Institute for Biology Tübingen, Tübingen, Germany

## Abstract

Zygnematophyceae are the closest algal relatives of land plants, yet their internal diversity has remained poorly sampled. This Primer explores a new study in PLOS Biology revealing deep radiations that reshape how we reconstruct the algal ancestry of plants.

## Teaser

Zygnematophyceae are the closest algal relatives of land plants, yet their internal diversity has remained poorly sampled. A new study in *PLOS Biology* reveals deep radiations that reshape how we reconstruct the algal ancestry of plants.

The closest algal relatives of land plants look deceptively simple. Zygnematophyceae include unicellular desmids and unbranched filamentous forms such as Spirogyra, yet phylogenomics has placed this morphologically reduced lineage as the sister group of land plants [1,2]. Their position as sister to land plants makes zygnematophytes particularly informative for reconstructing the algal ancestor of embryophytes and identifying features that evolved before the transition to land [3]. In a new study in this issue of *PLOS Biology*, Bierenbroodspot and colleagues greatly expand taxonomic sampling across Zygnematophyceae and uncover an unexpectedly deep genetic structure within the group ([4] Fig 1). Their results also show the limitations of reconstructing ancestral states from the relatively small number of zygnematophyte species that have been studied in detail.

**Fig 1:**
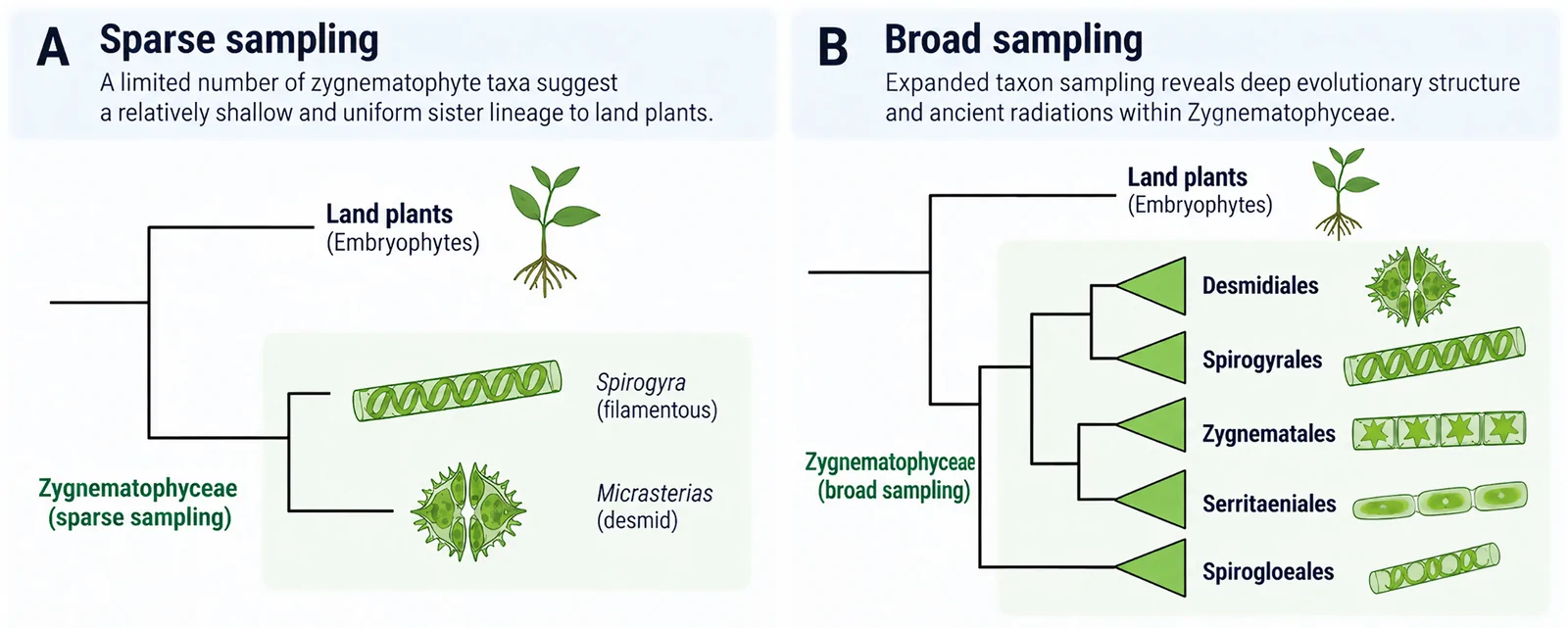
Broader taxon sampling reveals deep diversity within Zygnematophyceae. **(A)** Sparse sampling suggests a relatively shallow and morphologically uniform lineage. **(B)** Expanded sampling resolves five deeply diverging orders, represented here by characteristic morphologies, and provides a richer view of the algal sister lineage to land plants. Figure created using the ChatGPT image-generation tool (OpenAI), based on the author’s concept and phylogenetic framework, and subsequently reviewed and corrected for scientific accuracy.

The unexpected placement of Zygnematophyceae as the closest algal relatives of land plants has a long history. Earlier morphology-based scenarios often favoured more structurally elaborate streptophyte algae as the closest relatives of embryophytes. Molecular phylogenies overturned that expectation, repeatedly recovering Zygnematophyceae as sister to land plants [1,2]. This finding shifted attention towards the genomes of zygnematophytes, revealing that many molecular systems associated with land plant development, cell walls, signalling and stress responses have much deeper evolutionary origins than their comparatively simple morphology might suggest [5,6]. However, genomic and transcriptomic resources have remained concentrated in a small number of taxa. This is problematic because Zygnematophyceae are species-rich and morphologically diverse, and their internal branches may represent hundreds of millions of years of independent evolution.

Bierenbroodspot and colleagues address this sampling problem directly. They generated 43 new transcriptomes and combined these with existing data to analyse 104 zygnematophycean transcriptomes using 2,243 loci [4]. The resulting phylogenomic framework reveals pronounced genetic distances among major zygnematophyte lineages and signatures of ancient, rapid radiations. The large genetic distances between major lineages show that Zygnematophyceae represent a much deeper and more diverse evolutionary radiation than the relatively small number of species studied so far might suggest.

The new phylogeny also provides a different perspective on morphological evolution within the group. The authors recover a deep separation between Spirogyrales and their predominantly unicellular sister lineage Desmidiales, while much of the diversification within Spirogyrales appears to have occurred after substantial genetic divergence had already accumulated. This phylogenetic context is important for understanding the repeated appearance and disappearance of multicellular growth forms in streptophytes. Recent work increasingly points away from a simple evolutionary ladder running from unicellular algae through progressively more complex algal forms to land plants. Instead, multicellularity appears to have been evolutionarily dynamic, involving losses, reductions and secondary elaborations [7]. The new phylogeny provides a substantially better scaffold on which to map these transitions (Fig 1).

A particularly important implication concerns ancestral reconstruction. Because Zygnematophyceae are the sister lineage of embryophytes, traits shared between the two groups are frequently used to infer the biology of their last common ancestor. But the accuracy of such inference depends critically on sampling the phylogenetic breadth of the algal lineage. If a trait is absent from one familiar zygnematophyte but present in several deeply diverging lineages, its evolutionary interpretation changes dramatically. Conversely, features found in a single model species may represent relatively recent innovations rather than ancestral states. This highlights a more general problem in ancestral reconstruction: taxon sampling can strongly influence which traits are inferred to be ancestral

The expanded phylogenetic framework also opens important avenues for future research. While transcriptomes provide access to thousands of protein-coding genes, genome-scale sampling across the newly resolved diversity will enable more detailed investigation of gene-family evolution, genome architecture and regulatory innovation. Resolving deep and ancient radiations remains intrinsically challenging, particularly where short internodes and extensive sequence divergence generate conflicting phylogenetic signals. Rather than providing a final word on zygnematophycean relationships, this study establishes a substantially stronger framework for exploring their evolutionary history and, importantly, for reconstructing the biological innovations that preceded the emergence of land plants.

High-quality genomes from strategically selected species, combined with comparative cell biology and functional approaches, should make it possible to reconstruct when key cellular, developmental and regulatory innovations emerged and to distinguish ancestral features from lineage-specific innovations.

Studies of independently evolved multicellular lineages illustrate the power of such an approach. Brown algae, which evolved complex multicellularity independently of plants and animals, nevertheless show conserved developmental patterns across species with contrasting morphological complexity, revealing convergent constraints on multicellular development [8]. The newly resolved diversity of Zygnematophyceae now offers a similar opportunity: comparisons across its deep evolutionary breadth should help distinguish ancestral features from lineage-specific innovations on the path to land plants.

The study by Bierenbroodspot and colleagues makes clear how much of zygnematophyte evolutionary history has been missed by the limited taxonomic sampling available until now. By revealing the evolutionary depth of Zygnematophyceae, this study brings us closer to reconstructing the algal ancestor of land plants and the evolutionary changes that accompanied the transition to life on land.
